# MiR‐130b promotes the progression of oesophageal squamous cell carcinoma by targeting *SASH1*

**DOI:** 10.1111/jcmm.13887

**Published:** 2018-11-15

**Authors:** Yuxing Zhu, Yanni Ma, Honghua Peng, Lian Gong, Mengqin Xiao, Liang Xiang, Dong He, Ke Cao

**Affiliations:** ^1^ Department of Oncology The Third Xiangya Hospital of Central South University Changsha Hunan China; ^2^ Department of Respiration The Second People's Hospital of Hunan Province of Hunan University of Chinese Medicine Changsha Hunan China

**Keywords:** esophageal squamous cell carcinoma, MiR‐130b, *SASH1*

## Abstract

MiR‐130b and SAM and SH3 domain containing 1 (*SASH1*) play an important role in many types of human cancers. The aim of our research was to study their interactions in the process of the proliferation and aggressiveness of oesophageal squamous cell carcinoma (ESCC) cells. Microarray analysis was done to screen the differentially expressed genes in the ESCC tissues. miR‐130b and *SASH1* mRNA levels in the ESCC tissues and cells were detected by qRT‐PCR. Dual luciferase reporter system was used to verify the target relationship between miR‐130b and *SASH1*. The effects of miR‐130b on *SASH1* expression were explored by western blot in KYSE30 and TE1 cell lines. CCK‐8 assay, flow cytometry, Transwell, and wound healing assays were conducted to explore the effects of miR‐130b and *SASH1* in vitro. In addition, in vivo experiments were conducted to study the roles of miR‐130b and *SASH1*. miR‐130b was highly expressed, while *SASH1* was the opposite in both the ESCC tissues and cells. The expression of *SASH1* was inhibited by the direct binding of miR‐130b. The inhibition of miR‐130b reduced the proliferation and aggressiveness of ESCC cells, while it also induced apoptosis and cell cycle arrest in the ESCC cells by suppressing *SASH1*. The in vivo assay suggested that the overexpression of miR‐130b promoted the growth of ESCC tumours. MiR‐130b was up‐regulated in the ESCC tumour tissues and cells, acting as a tumour promoter. A stimulating effect was demonstrated on ESCC cell growth and aggressiveness by suppressing *SASH1,* which is an anti‐oncogene.

## 1 INTRODUCTION

Oesophageal cancer is the eighth most common cancer and the sixth most common cause of cancer death worldwide.^1^ A total of 70% of all oesophageal cancer worldwide occurs in China, of which 90% of cases are oesophageal squamous cell carcinoma (ESCC).^2^ Most ESCC patients are diagnosed at the advanced stage,^3^ and the management of ESCC is difficult because of early aggressive metastasis to lymph nodes and organs, which result in the poor prognosis.^4^ Recently, tremendous efforts have focused on identifying specific molecular markers associated with the progression of ESCC to better understand the etiology of the disease to improve the diagnosis and treatment of ESCC.^5^

MicroRNAs (miRNAs) are short (19–25 nucleotides), highly conserved, single‐stranded, noncoding RNAs.^6^ The differential expression of the miRNAs between tumour tissue and normal tissue is observed in various cancer types, suggesting a possible link between miRNA expression and the development of cancer.^7^ MiRNAs regulate both oncogenes and tumour suppressor genes.^8^ This highlights the potential of using the expression profiles of specific miRNAs for cancer diagnosis or therapy.

MiR‐130b, located at the 22q11 locus,^9^ plays an oncogenic role in many types of human cancers, including gastric cancer, endometrial cancer^10^, ^11^ and ESCC. MiR‐130b is significantly up‐regulated and promotes the proliferation and aggressiveness of cell lines by repressing phosphatase and tensin homolog (*PTEN*) expression.^12^ MiR‐130b promotes cell growth and self‐renewal via tumour protein 53‐induced nuclear protein 1 (*TP53INP1*). Recently, it was reported that the down‐regulation of miR‐130b in ovarian cancer is associated with progression, multidrug resistance, and poor histological differentiation.^13^ Lai et al reported that miR‐130b contributed to tumourigenesis and progression by regulating the tumour suppressor runt related transcription factor 3 (*RUNX3*) in gastric cancer.^11^ Previous studies show that miR‐130b may be a tumour and metastasis facilitator. However, the biological role of miR‐130b in ESCC remains unclear.

SAM and SH3 domain containing 1 (*SASH1*), a member of the SLY family of signal adapter proteins, is located at the chromosomal locus 6q24.3 and is 279,746 base pairs in length. The expression products play an important role in intracellular signalling by mediating protein‐protein interactions. For example, *SASH1* encodes a scaffold protein involved in the toll‐like receptor 4 signalling pathway that may stimulate cytokine production and endothelial cell migration in response to invading pathogens. The encoded protein has also been described as a potential tumour suppressor that negatively regulates the proliferation and aggressiveness of multiple cancer cells. He et al reported that the overexpression of *SASH1* inhibits proliferation, invasion and epithelial‐mesenchymal transition (EMT) in hepatocarcinoma cells.^14^ *SASH1* overexpression in lung cancer cells also inhibits the migration/invasion and the protein expression of cyclin D1, matrix metalloproteinase‐1 (MMP‐1), and MMP‐2.^15^ However, the tumourigenic roles and mechanisms underlying the down‐regulation of *SASH1* in ESCC are still largely unknown.

In this study, we analysed miR‐130b and *SASH1* expression level in ESCC tissues and cells. The target relationship between miR‐130b and *SASH1* was studied. We intended to explore the potential therapeutic value of miR‐130b and *SASH1* in order to provide more support and help improve the survival of ESCC patients.

## 2 MATERIALS AND METHODS

### 2.1 Clinical samples

Clinical ESCC and the matched adjacent normal tissue samples were collected from 20 patients with ESCC who underwent oesophagectomy at the Third Xiangya Hospital of Central South University during June 2016 and July 2017. The patients were pathologically diagnosed with ESCC and were not subjected to preoperative chemotherapy and/or radiotherapy. All the experiments were ratified by the Ethics Committee of the Third Xiangya Hospital of Central South University, and informed consents were provided by all the subjects.

### 2.2 Microarray analysis

The datasets GSE55857, GSE97051 and GSE23400 were downloaded from the National Center of Biotechnology Information Gene Expression Omnibus database. GSE55857 was based on the GPL14613 platform of the Affymetrix Multispecies miRNA‐2 Array. A total of 216 samples were divided into two groups, including the ESCC tissue samples (n = 108) and the normal samples (n = 108). GSE97051 was based on the GPL20115 platform of the Agilent‐067406 Human CBC lncRNA + mRNA microarray V4.0. A total of 14 samples were divided into two groups, including the ESCC tissue samples (n = 7) and the normal samples (n = 7). GSE23400 was based on the GPL96 platform of the Affymetrix Human Genome U133A Array. A total of 20 samples were divided into two groups, including the ESCC tissue samples (n = 10) and the normal samples (n = 10). An analysis of the expression profile data was performed using R 3.4.1 (https://www.r-project.org/) with the Limma program. The screening thresholds were the adjusted *P*‐value <0.05 and the log_2_(fold change) >1.

### 2.3 Cell lines and small molecules

The ESCC cell lines (KYSE30, KYSE150, TE1 and EC9706) were cultured in RPMI‐1640/F‐12 medium (Invitrogen, Waltham, MA, USA) with 10% foetal bovine serum (FBS; HyClone, Logan, UT, USA), penicillin (100 U/ml) and streptomycin (100 mg/mL). The immortalized human oesophageal epithelial cell line SHEE was cultured in 90% DMEM with 10% FBS. All the cell lines were purchased from the BeNa Culture Collection (Shanghai, China). Cell transfection was conducted using the Lipofectamine 3000 transfection reagent (Invitrogen), following the manufacturer's manual. The miR‐130b mimics (50 nM), agomiR‐130b (for in vivo experiment, 100 nM), a miR‐130b inhibitor (50 nM) and a blank plasmid transfection group (negative control) (50 nM) were synthesized by GenePharma (Shanghai, China), and their sequences are listed in Table S1.

### 2.4 qRT‐PCR

The total RNA from the clinical specimens and ESCC cells was extracted using TRIzol and was reverse transcribed into cDNA using the RevertAid First Strand cDNA Synthesis Kit (Thermo Fisher Scientific, Waltham, MA, USA). Real‐time PCR was performed using the SYBR Green Premix PCR Master Mix (Roche, Mannheim, Germany). All the procedures followed the manufacturer's manuals. The relative expressions of miR‐130b and *SASH1* were calculated by the 2^−∆∆C*t*^ method after being normalized to U6 small nuclear RNA and glyceraldehyde‐3‐phosphate dehydrogenase, respectively. The sequences of the PCR primers used are listed in Table S2.

### 2.5 Western blot

The cells were lysed with RIPA lysis buffer, including 0.1% PMSF, on ice for 20 minutes. The proteins were separated using 12% SDS‐PAGE and were electroblotted to a PVDF membrane. The membranes were blocked in 5% nonfat milk at 4°C overnight. The next day, the membranes were incubated with anti‐*SASH1* (rabbit polyclonal to *SASH1*, 1:500, ab110776) and anti‐β‐actin (rabbit polyclonal to β‐actin, 1:1000, ab8227) for 2 hours. Then, a secondary HRP‐labelled goat anti‐rabbit IgG antibody (1:50 000, ab205718) was incubated with the membrane for 0.5 hour. All the antibodies were purchased from Abcam (Eugene, OR, USA).

### 2.6 CCK‐8 assay

Cell proliferation was measured by a Cell Counting Kit‐8 (CCK‐8; Dojindo Laboratories, Kumamoto, Japan). The experiment was conducted in a 96‐well plate 24 hours after transfection. At each indicated time‐point, 10 μL of CCK‐8 was added to each well for a 1 hour incubation. The optical absorbance at 450 nm was detected in a plate reader.

### 2.7 Wound healing assay

The experiment was conducted in a 96‐well plate 24 hours after transfection. The ESCC cell lines KYSE30 and TE1, which were selected out from the previous experiments were utilized. At 24 hours post‐transfection with 100 nM miRNA mimics, a scratch was made through the centre of each well using a 1000 μL pipette tip, creating an open “wound” that was clear. The distance of migration into the open area was measured at 24 hours postscratching.

### 2.8 Transwell assay

A total of 50 μL of Matrigel (0.2 μg/μL, diluted in RPMI‐1640/F‐12 medium) was added to the upper chamber of the Transwell and was air‐dried at 4°C. The KYSE30 and TE1 cells were added to the upper chamber of the Transwell, and 900 μL of RPMI‐1640/F‐12 with 5% FBS was added to the lower chamber. Ten random fields of every view for each sample were observed.

### 2.9 Flow cytometry assay

Cell cycle was analysed by flow cytometry (FCM) using a cell cycle detection kit (KeyGEN Biotech, Nanjing, China). PE‐Texas‐Red was used to dye the cells. Apoptosis was determined using the Annexin V‐FITC apoptosis detection kit (KeyGEN Biotech, Nanjing, China). The KYSE30 and TE1 cells were double‐stained with propidium iodide and Annexin V‐FITC before they underwent the FCM analysis.

### 2.10 Dual luciferase reporter gene assay

The target gene of hsa‐miR‐130b was predicted by bioinformatics prediction software. The target fragment was subcloned into a pMirTarget vector downstream of the firefly luciferase gene to generate the recombinant luciferase reporter vectors pMIR‐*SASH1*. The mutagenesis of the seed region was achieved by overlapping PCR to generate the mutated *SASH1* 3′UTR. All the inserted sequences were verified by direct DNA sequencing. The recombinant plasmids were cotransfected into the KYSE30 cells with Lipofectamine 3000 (Invitrogen).

### 2.11 Xenograft assay

The animal experiments were approved by the committee of the Use of Animal Care in the Third Xiangya Hospital of Central South University. BALB/c mice (4‐5 weeks old, 18‐20 g) were purchased from the Animal Center of Fudan University (Shanghai, China). A total of 1 × 10^6^ KYSE30 cells was injected into the left dorsal sides of the mice. Approximately 7 days later, when the grafted tumours were apparent (approximately 50 mm^3^), 100 μL of vectors (NC, blank plasmids), agomiR‐130b, and *SASH1* overexpression plasmids were injected into the inoculation site of every mouse every 2 weeks over the next 30 days. The tumour volumes, (length × width^2^)/2, were measured every week, and the tumour weights were measured after 4 weeks when the mice were killed.

### 2.12 Statistical analysis

The experimental data were analysed using GraphPad Prism 6.0 Software (GraphPad Inc., California, CA, USA). All the experiments were done at least three times. The data are expressed as the average ± SD. A Student's *t* test (2 groups) and a one‐way analysis of variance (multiple groups) were conducted to analyse the differences.

## 3 RESULTS

### 3.1 MiR‐130b is overexpressed in ESCC tissues and cell lines

**Figure 1:**
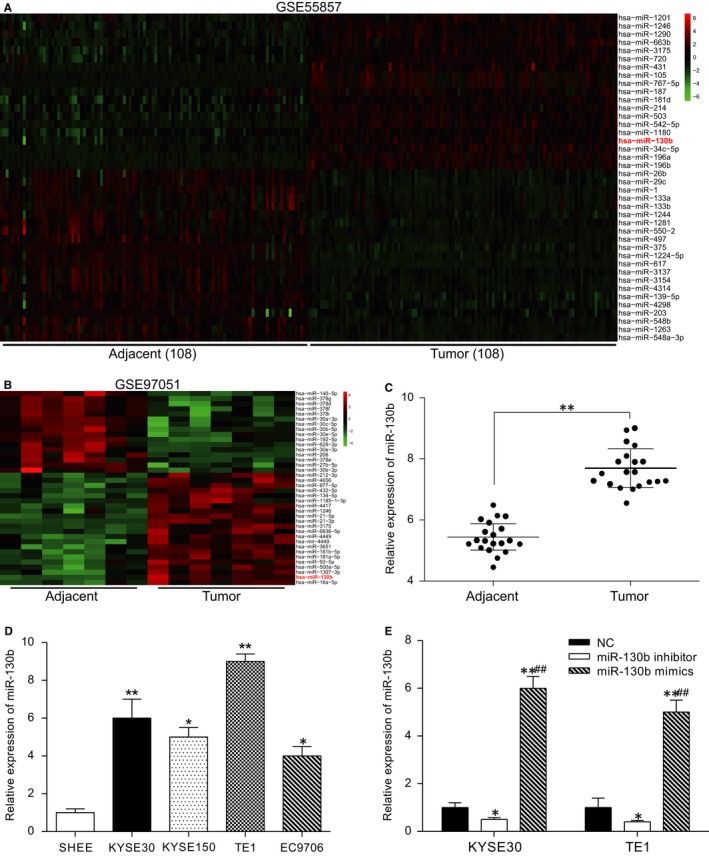
MiR‐130b is highly expressed in ESCC tissues and cell lines. (A) Data from GSE55857: the differentially expressed miRNAs are illustrated in a heatmap. MiR‐130b was part of the overexpression category. (B) Data from GSE97051: the differentially expressed miRNAs are illustrated in a heatmap. MiR‐130b was part of the overexpression category. (C) The qRT‐PCR results showed the overexpression of miR‐130b in the tumour tissues. **P < 0.01, compared with the adjacent tissues. (D) The expression level of miR‐130b in the ESCC cell lines (KYSE30, KYSE150, TE1, EC9706 and SHEE) was examined by qRT‐PCR. *P < 0.05, **P < 0.01, compared with the SHEE cell line. (E) The expression level of miR‐130b in the KYSE30 and TE1 cells transfected with the miR‐130b mimics or the miR‐130b inhibitor was determined using qRT‐PCR. *P < 0.05, **P < 0.01, compared with the NC group; ^##^ *P* < 0.01, compared with the miR‐130b inhibitor group

The bioinformatics analysis based on GSE5857 (Figure 1A) and GSE97051 (Figure 1B) showed that miR‐130b was overexpressed in the ESCC tissues. To validate this result, a qRT‐PCR was carried out. The experimental analysis (qRT‐PCR) results also showed that miR‐130b was overexpressed in the ESCC tissues (Figure 1C). Parallelly, the expression of miR‐130b in the human ESCC cell lines (KYSE30, KYSE150, TE1, and EC9706) was remarkably higher compared with that in the SHEE cells (*P* < 0.05). The KYSE30 and TE1 cell lines showed the highest miR‐130b levels among all the cell lines (*P* < 0.01, Figure 1D). Hence, the KYSE30 and TE1 cells were chosen for the subsequent experiments. The expression of miR‐130b was significantly higher in the miR‐130b mimics groups, while the expression was lower in the inhibitor group compared with the NC group. This result indicated that the transfection efficiency was then verified in the TE1 cell line (*P* < 0.05, Figure 1E).

### 3.2 MiR‐130b promotes the proliferation, migration, and invasion of the ESCC cells

**Figure 2:**
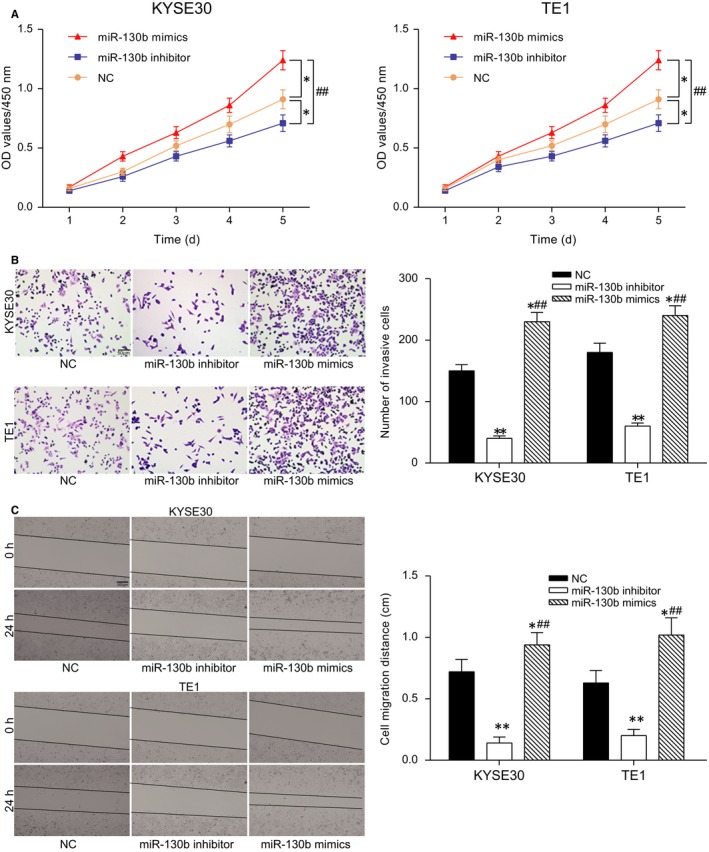
MiR‐130b promotes the proliferation, migration and invasion of ESCC cells. (A) The CCK‐8 assay results showed the proliferation of the KYSE30 and TE1 cells for the different groups. *P < 0.05, compared with the NC group; ^##^ *P* < 0.01, compared with the miR‐130b inhibitor group. (B) The Transwell assay results demonstrated the cell invasion of the different groups. The quantification results are shown in the histogram. *P < 0.05, **P < 0.01, compared with the NC group; ^##^ *P* < 0.01, compared with the miR‐130b inhibitor group. (C) The wound healing assay results demonstrated the cell migration distance of the different groups. The quantification results are shown in the histogram. *P < 0.05, **P < 0.01, compared with the NC group; ^##^ *P* < 0.01, compared with the miR‐130b inhibitor group

The CCK‐8 assay results displayed that the cell viability of the KYSE30 and TE1 cells was suppressed in the miR‐130b inhibitor group but was promoted in the miR‐130b mimics group (*P* < 0.05, Figure 2A). Mimic delivery increased the invading cell number, and the inhibitor delivery reduced the invading cell number (*P* < 0.05, Figure 2B). At 24 hours, the wound closure was longer in the miR‐130b inhibitor group (*P* < 0.01) and was shorter in the miR‐130b mimics group than the NC group (*P* < 0.05, Figure 2C). The above results indicated that miR‐130b promoted the progression of proliferation, migration, and invasion in the ESCC cells.

### 3.3 MiR‐130b promotes cell cycle progression and apoptosis in ESCC cells

**Figure 3:**
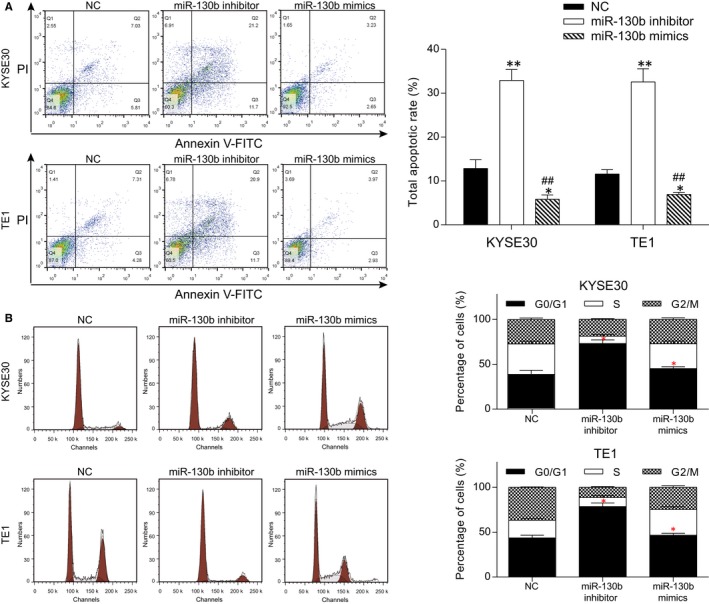
MiR‐130b inhibits ESCC cell cycle blockade and apoptosis. Flow cytometry was used to assess apoptosis and cell cycle. (A) The apoptosis rates of the cells in the different groups are shown. Propidium iodide (PI) and annexin V‐FITC were the dyes. The histogram shows the apoptosis statistics. (B) The cell cycle distribution of the cells in the different groups is shown. A histogram was given. *P < 0.05, **P < 0.01, compared with the NC group; ^##^ *P* < 0.01, compared with the miR‐130b inhibitor group

The apoptosis rate of the KYSE30 and TE1 cells was considerably higher after the transfection with the miR‐130b inhibitor (*P* < 0.01) and was significantly lower after transfection with the miR‐130b mimics compared with the NC group (*P* < 0.05, Figure 3A). In terms of the cell cycle distribution of the two cell lines, compared with the NC group, there were significantly more cells in the G0/G1 phase in the miR‐130b inhibitor group and fewer in the miR‐130b mimics group (*P* < 0.05, Figure 3B). The experiments above revealed that miR‐130b promoted cell cycle progression as well as the apoptosis rate in the ESCC cells.

### 3.4 *SASH1* is a direct target of miR‐130b

**Figure 4:**
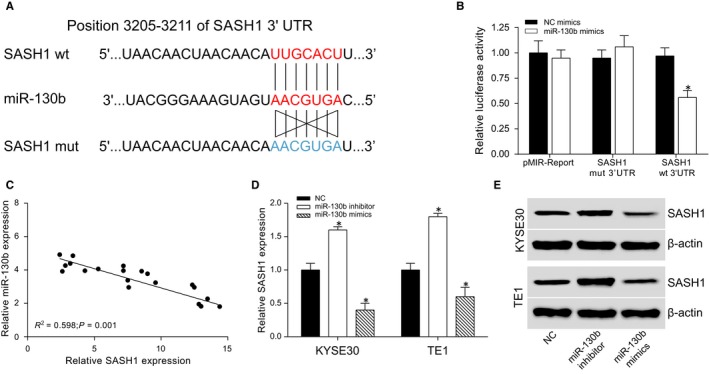
*SASH1* is a direct target of miR‐130b. (A) The miR‐130b, wild‐type 3′UTR and mutated 3′UTR sequences of *SASH1* were obtained from TargetScan and are illustrated. (B) The dual luciferase reporter gene assay results showed a direct binding interaction between miR‐130b and *SASH1*. *P < 0.05, compared with the NC mimics group. (C) The expression of miR‐130b and *SASH1* were negatively correlated. (D) The qRT‐PCR results showed the mRNA levels of *SASH1* in the KYSE30 and TE1 cells. *P < 0.05, compared with the NC group. (E) The protein expression levels of *SASH1* in the KYSE30 and TE1 cells in the different groups

*SASH1* mRNA was under‐expressed in the ESCC tissues (*P* < 0.01, Figure S1A and B). In addition, the protein expression of *SASH1* in the ESCC tissues was also low (*P* < 0.01, Figure S1C and D). The wild‐type and mutated *SASH1* 3′UTR of interest, as well as the sequence of miR‐130b, which contains the binding site of wild‐type *SASH1* 3′UTR, are illustrated (Figure 4A). MiR‐130b was directly bound to the 3′UTR seed sequence region of the *SASH1* gene and inhibited its expression (Figure 4B). The expression of miR‐130b was negatively correlated with *SASH1* expression (Figure 4C). The Western blot assay indicated that the overexpression of miR‐130b significantly decreased *SASH1* expression compared with the control group. In contrast, the down‐regulation of miR‐130b increased *SASH1* expression (Figure 4D and E).

### 3.5 MiR‐130b promotes ESCC cell proliferation and aggressiveness by suppressing *SASH1*

**Figure 5:**
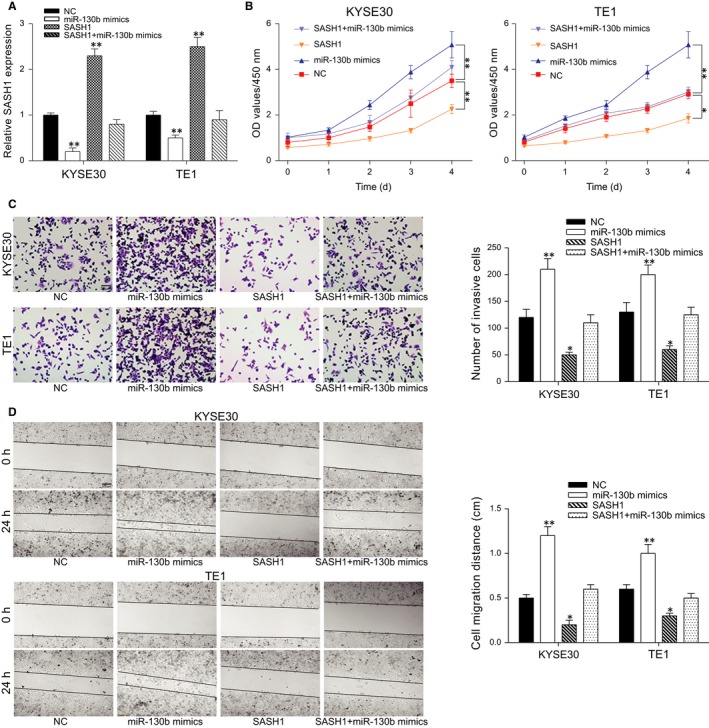
MiR‐130b promotes the proliferation, migration and invasion of ESCC cells by targeting *SASH1*. (A) The relative mRNA levels of *SASH1* in the different groups for the KYSE30 and TE1 cells were examined using qRT‐PCR and are presented in the histogram. (B) The CCK‐8 assay results are shown, demonstrating the proliferation of the KYSE30 and TE1 cells in the different groups. (C) The invasiveness results are shown. A histogram was also shown to demonstrate the statistics. (D) The migration test results are shown. A histogram is also given. *P < 0.05, **P < 0.01, compared with the NC group

The overexpression of miR‐130b significantly promotes cell proliferation and aggressiveness as described above. In contrast, the overexpression of *SASH1* significantly suppressed the proliferation and aggressiveness of both cell lines (*P* < 0.05, Figure 5). We first confirmed the efficiency of the miR‐130b mimics and the *SASH1* overexpression plasmids in the KYSE30 and TE1 cells (Figure 5A). The proliferation and aggressiveness of the cells in the *SASH1*+ miR‐130b mimics group were insignificantly less compared with the NC group (*P* > 0.05, Figure 5B and D). All of these results indicated that miR‐130b inhibited the proliferation and invasion of ESCC cells by targeting *SASH1*.

### 3.6 MiR‐130b hinders ESCC cell cycle blockade and apoptosis by targeting *SASH1*

**Figure 6:**
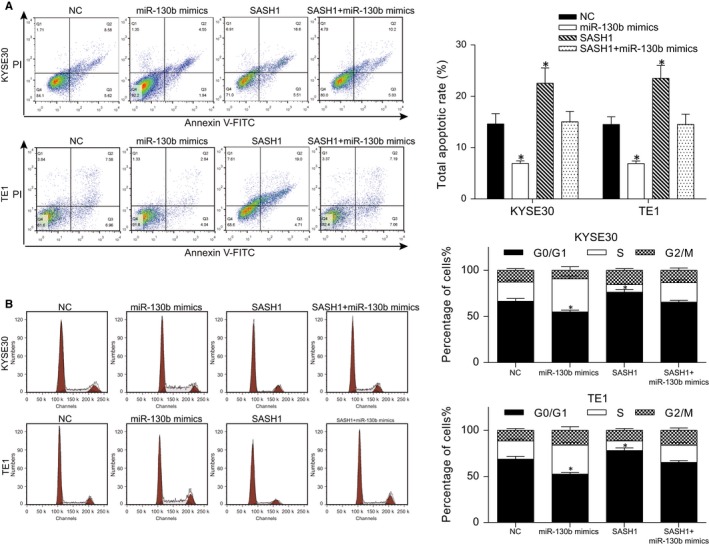
MiR‐130b inhibits cell cycle blockade and apoptosis in the ESCC cells by targeting *SASH1*. Apoptosis and cell cycle were determined using flow cytometry. (A) The apoptosis rate of the cells in the different groups are shown. Propidium iodide (PI) and annexin V‐FITC were the dyes that were used. The histogram shows the apoptosis rate. (B) The cell cycle distribution of the cells in the different groups are shown. A histogram is given. *P < 0.05, compared with the NC group

The apoptosis rate was decreased in both cell lines after miR‐130b transfection, whereas the overexpression of *SASH1* promoted it (Figure 6A). The overexpression of miR‐130b resulted in fewer G0/G1 phase blocked cells. Whereas *SASH1* overexpression caused more cells to be blocked in the G0/G1 phase (Figure 6B). It is notable that the apoptosis rate in *SASH1*+ miR‐130b mimics group was not significantly different compared with those in the NC group; meanwhile, the cell cycle assays also indicated that the cell cycle in this group showed no significant difference compared with the NC group, which suggested that miR‐130b regulated the cell cycle by targeting *SASH1* in the ESCC cell lines.

### 3.7 MiR‐130b promotes ESCC tumour growth in vivo

**Figure 7:**
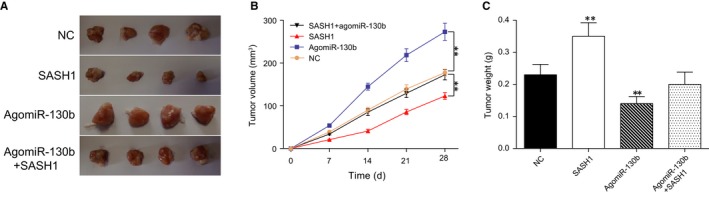
MiR‐130b facilitates the proliferation of ESCC cells in vivo. (A) The tumours that were harvested from the nude mice were photographed and are presented here. (B) The tumour volumes of the mice in the different groups are illustrated in the line diagram. (C) The tumour weights of the mice in the different groups are illustrated in the histogram. **P < 0.01, compared with the NC group

The tumours of the xenograft mice models are shown in Figure 7A. Generally, the tumour size and volume in the agomiR‐130b group were the largest, whereas those in the *SASH1* group were the smallest (*P* < 0.01, Figure 7B). The continuous delivery of agomiR‐130b led to a greater tumour weight, whereas the continuous delivery of the *SASH1* overexpression plasmids yielded a reduced tumour weight (*P* < 0.01, Figure 7C). All together, these data indicated that miR‐130b promoted the growth of ESCC tumours by suppressing the anti‐oncogene *SASH1* in vivo.

## 4 DISCUSSION

MiR‐130b was overexpressed in both the ESCC tissues and cell lines through our assays. The overexpression of miR‐130b promoted proliferation and aggressiveness, as well as apoptosis, in the ESCC cells in vitro by directly binding to *SASH1*. The knockdown of miR‐130b reduced ESCC cell proliferation and aggressiveness. In addition, the overexpression of miR‐130b enhanced tumour growth in vivo by suppressing *SASH1*.

The ectopic expression of miRNAs is closely associated with human cancer development and progression. The aberrant expression of miR‐130b facilitates the progression of gastric cancer^11^ and endometrial cancer.^10^ However, the underexpression of miR‐130b also occurs in ovarian cancer.^13^ We found that miR‐130b was up‐regulated in ESCC, which was consistent with a previous study of miR‐130b in ESCC,^8^ suggesting that the ectopic expression of miR‐130b might have specific functions, depending on the type of malignancy. In addition, consistent with the previous findings of miR‐130b in ESCC, miR‐130b performed as a tumour promotor in our current study.

A single miRNA can target multiple mRNAs and affect a variety of cellular pathways.^16^ To date, several target genes of miR‐130b have been identified. In hepatocellular carcinoma (HCC) cells, researchers found that miR‐130b promotes cell aggressiveness and EMT by targeting PPAR‐γ.^17^, ^18^ However, Lin et al found that the overexpression of miR‐130b caused a remarkable suppression of HCC cell aggressiveness by inhibiting *IRF1*.^19^ In addition, miR‐130b may have an effect on the tumourigenesis of cold and benign thyroid nodules by regulating proliferation and apoptosis and the cell cycle through cyclin D1.^20^ TGF‐β1 acts through miR‐130b to promote integrin α5 expression, resulting in the enhanced migration of clear renal carcinoma cells.^21^

*SASH1* plays an important role in cancer initiation, development, and metastasis. Several studies report that *SASH1* is down‐regulated in tumours and that this expression is correlated with tumour grade and prognosis. *SASH1* is down‐regulated in a number of cancers, including HCC,^14^ breast cancer,^22^ gastric cancer,^23^ *etc*. Consistently, in this study, *SASH1* was underexpressed in the ESCC cell lines, suggesting that *SASH1* might be a candidate tumour suppressor gene in ESCC. Plus, we confirmed that *SASH1* was a direct target of miR‐130b. Taken together, the miR‐130b/*SASH1* axis works closely during ESCC tumourigenesis. MiR‐130b may facilitate ESCC progression by inhibiting *SASH1* expression.

We revealed a novel function of miR‐130b in promoting proliferation and progression by directly targeting the 3′UTR of *SASH1*. Nevertheless, some limitations exist in this study. The sophisticated molecular mechanism of the down‐regulation of miR‐130b and the target relationship between miR‐130b and *SASH1* in ESCC cell still need further investigation. Moreover, miR‐130b, as a novel biomarker and target for treatment, remains to be further explored at the clinical level.

In summary, miR‐130b was up‐regulated in ESCC tumour tissues and cells, acting as a tumour promotor. MiR‐130b demonstrated a stimulating effect on ESCC cell growth and aggressiveness by suppressing *SASH1*. It also promoted tumour formation in vivo by targeting *SASH1*. Our findings suggested that miR‐130b might contribute to the development of a miRNA‐based therapy for ESCC.

## CONFLICT OF INTEREST

The authors confirm that there are no conflicts of interest.
