# Supplementary figures and images for: MiR‐130b promotes the progression of oesophageal squamous cell carcinoma by targeting SASH1
Source: J Cell Mol Med. 2018 Nov 15;23(1):93–103. doi: 10.1111/jcmm.13887 (PMC6307769; doi:10.1111/jcmm.13887)

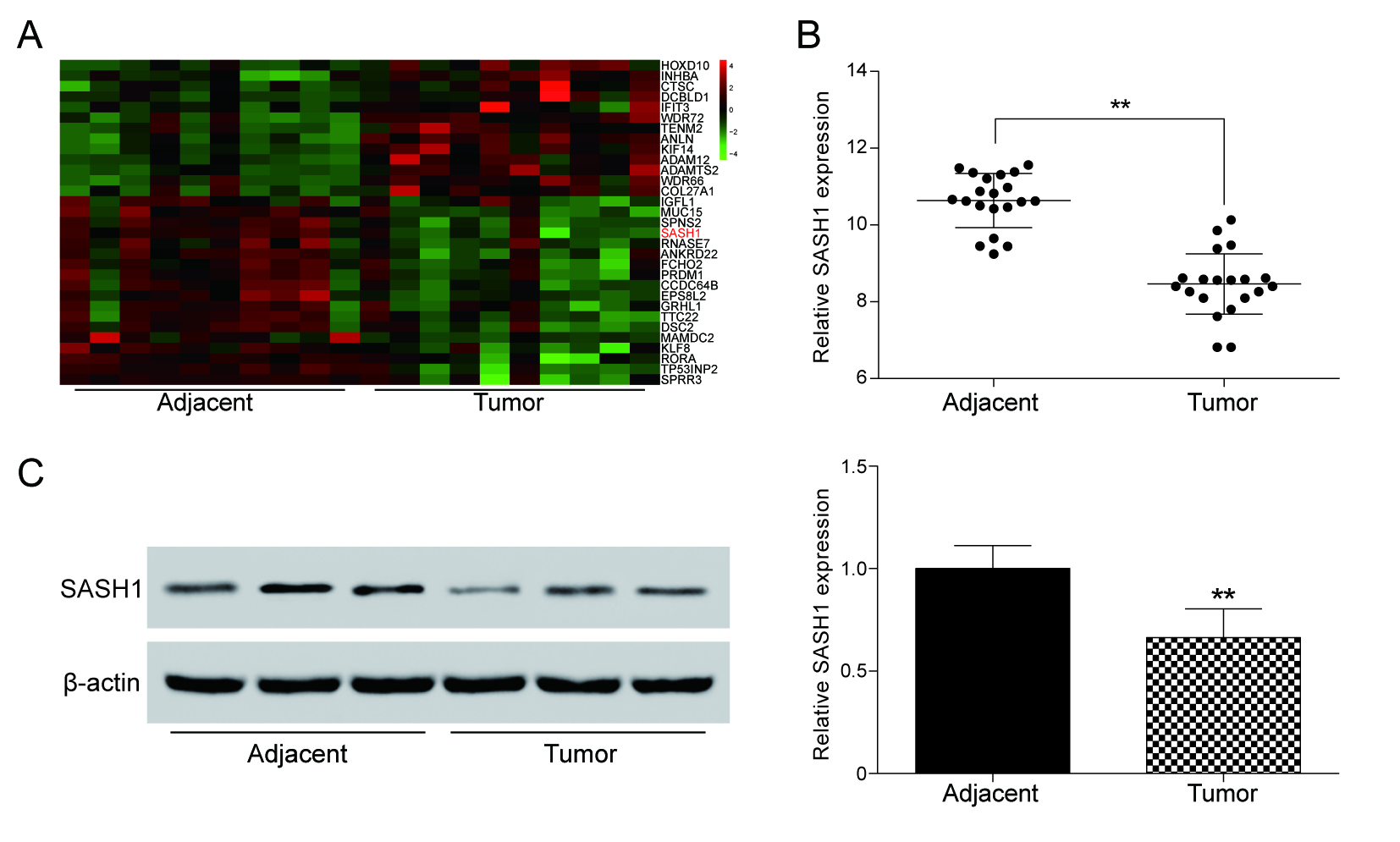

Supplement: Supplementary file 1 [file JCMM-23-93-s001.tif]
