# Supplementary material for: MiR‐130b promotes the progression of oesophageal squamous cell carcinoma by targeting SASH1
Source: J Cell Mol Med. 2018 Nov 15;23(1):93–103. doi: 10.1111/jcmm.13887 (PMC6307769; doi:10.1111/jcmm.13887)
Supplement: Supplementary file 2 [file JCMM-23-93-s002.docx]

**Table S1. Sequences of miRNA oligonucleotides**

| oligonucleotides | Sequence 5’-3’ |
| --- | --- |
| miR-130b mimics/agomiR-130b | CAGUGCAAUGAUGAAAGGGCAU |
| miR-130b inhibitor | AUGCCCUUUCAUCAUUGCACUG |
| Negative control | UUGUACUACACAAAAGUACUG |
