# Supplementary material for: MiR‐130b promotes the progression of oesophageal squamous cell carcinoma by targeting SASH1
Source: J Cell Mol Med. 2018 Nov 15;23(1):93–103. doi: 10.1111/jcmm.13887 (PMC6307769; doi:10.1111/jcmm.13887)
Supplement: Supplementary file 3 [file JCMM-23-93-s003.docx]

**Table S2. PCR Primer sequences**

| Gene | Sequence 5’-3’ |
| --- | --- |
| miR-130b forward | ACACTCTTTCCCTGTTGCACT |
| miR-130b reverse | GACCTGACCGATGCCCTTTC |
| U6 forward | CTCGCTTCGGCAGCACATA |
| U6 reverse | AACGATTCACGAATTTGCGT |
| SASH1 forward | CGGGAAAGCGTCAAGTCGGA |
| SASH1 reverse | ATCTCCTTTCTCTTGAGCTTGAG |
| GAPDH forward | GAAGGTGAAGGTCGGAGTC |
| GAPDH reverse | GAAGATGGTGATGGGATTTC |
